# Metformin Associated Lactic Acidosis in the Intensive Care Unit: A Rare Condition Mimicking Sepsis

**DOI:** 10.7759/cureus.9119

**Published:** 2020-07-10

**Authors:** Selin Sendil, Keerthi Yarlagadda, Halimat Lawal, Vinod Nookala, Hiren Shingala

**Affiliations:** 1 Internal Medicine, University of Pittsburgh Medical Center (UPMC) Pinnacle, Harrisburg, USA; 2 Internal Medicine, Community Medical Center, Toms River, USA; 3 Critical Care Medicine, University of Pittsburgh Medical Center (UPMC) Pinnacle, Harrisburg, USA

**Keywords:** metformin associated lactic acidosis, mala, metformin, renal failure, crrt

## Abstract

Metformin-associated lactic acidosis (MALA) is a rare but serious complication of metformin use, associated with high mortality. MALA can occur any time a patient on metformin suffers disruption in renal function resulting in the accumulation of metformin.

A 63-year-old man with a history of non-insulin-dependent type 2 diabetes mellitus, alcohol abuse, and hypothyroidism was brought to the emergency department with altered mental status, nausea, vomiting, and abdominal pain. He was found to be in respiratory distress, was hypotensive and hypoglycemic (48 mg/dL), and required emergent intubation. Blood work was significant for pH<6.69, undetectable bicarbonate, anion gap 37.2 mEq/L, lactate >12 mmol/L, creatinine 15.95 mg/dL, blood urea nitrogen (BUN) 112 mg/dL, glomerular filtration rate (GFR), 3 ml/min/1.73sqm, and potassium 7 mmol/L. He suffered cardiac arrest, underwent cardiopulmonary resuscitation (CPR), and was admitted to the intensive care unit (ICU) where he required multiple vasopressors, bicarbonate infusion, and bicarbonate pushes. He was started on continuous renal replacement therapy with a high flux membrane. A high dose of pre- and post- filter fluids was used to improve conductive clearance. His pH corrected to normal in less than 24 hours, and hemodialysis was initiated the following day for a total of four days. Head/chest/abdomen/pelvis CT, urine, and blood cultures did not reveal any pathology that would explain lactic acidosis. The patient’s dose of metformin was 1 gr twice daily and sitagliptin, 100 mg daily. Blood metformin that had been tested on admission was 29 mcg/ml (therapeutic range, 1-2 mcg/ml). Methanol, ethanol, ethylene glycol, propylene glycol, and isopropanol levels were negative. He had been started on lisinopril 5 mg and amitriptyline 25 mg four weeks prior to admission and had normal creatinine at that time. He was discharged to an acute rehabilitation facility on day seven of hospitalization.

MALA generally presents with nausea, vomiting, and fatigue-often mimicking sepsis. It is possible that our patient progressively developed alcoholic ketoacidosis and acute renal failure from dehydration and excessive drinking in the setting of newly started Angiotensin-converting-enzyme (ACE) inhibitor. Recommendations for the optimal treatment of MALA mostly depend on expert opinion and case reports. Treatment is restricted to supportive measures, although hemodialysis may offer a protective effect. Our case demonstrates that even in extreme cases of MALA, prompt and adequate supportive measures can produce a favorable outcome.

## Introduction

Metformin-associated lactic acidosis (MALA) is a rare but potentially fatal complication of metformin use with a 30-50% mortality rate [1]. MALA can occur any time a patient on metformin suffers a disruption in renal function resulting in an accumulation of metformin. Here, we present a case of a 63-year-old male with non-insulin-dependent type II diabetes mellitus (NIDDM) on metformin who developed severe lactic acidosis, profound acidemia, significant electrolyte abnormalities, and ultimately cardiac arrest in the setting of acute kidney injury (AKI). 

## Case presentation

A 63-year-old Caucasian male with a past medical history of NIDDM, alcohol use disorder, anxiety, and hypothyroidism was brought to the emergency department (ED) via emergency medical services (EMS) with altered mental status. He had reportedly been having nausea, vomiting, and abdominal pain for one week, and also developed progressively worsening change in vision for about 48 hours. Initially, EMS found his blood glucose 48 mg/dL and administered intravenous (IV) dextrose. Upon arrival at the ED, he was found to be in respiratory distress with labored breathing and respiratory rate of 22/minute. His heart rate was 63 beats per minute, blood pressure 47/33 mmHg, temperature 92.6 °F (rectal temperature). The patient required emergent intubation in the ED. His initial blood work was significant for pH<6.69 (could not be calculated by our institution's blood gas analyzer), undetectable bicarbonate (could not be calculated by our institution's blood gas analyzer), anion gap 37.2 mEq/L, lactate >12 mmol/L, creatinine 15.95 mg/dL, blood urea nitrogen (BUN) 112 mg/dL, glomerular filtration rate (GFR) 3 ml/min/1.73sqm, phosphorus 17.7 mmol/L, potassium 7 mmol/L (Table 1).

**Table 1:** The patient's BMP from a month ago and the BMP obtained in the ED BMP was unremarkable a month ago when started on lisinopril. BMP: basic metabolic panel; BUN: blood urea nitrogen; GFR: glomerular filtration rate; ALP: alkaline phosphatase; ALT: alanine aminotransferase; AST: aspartate aminotransferase; ED: emergency department

Parameters	BMP a month prior to presentation	BMP after initial stabilization
Sodium (mmol/L)	140	131
Potassium (mmol/L)	4.4	7
Chloride (mmol/L)	105	89
CO_2_ (mmol/L)	25.9	4.8
Anion Gap (mEq/L)	9.1	37.2
BUN (mg/dL)	14	112
Creatinine (mg/dL)	0.81	15.95
GFR (ml/min/1.73sqm)	>90	3
Phosphorus (mg/dL)		17.7
Glucose (mg/dL)	119	138
Calcium (mg/dL)	9.3	7.8
ALP (U/L)	123	72
ALT (U/L)	14	17
AST (U/L)	16	21
Total Bilirubin (mg/dL)	0.3	0.3
Total Protein (g/dL)	6.7	5.1
Albumin (g/dL)	3.9	3

His white blood cell count (WBC) was 9.5 K/µL, neutrophils 50%, band 6% (Table 2), activated partial thromboplastin time 79.3 seconds, and international normalized ratio 1.1. He developed cardiac arrest in the ED, received brief cardiopulmonary resuscitation (CPR) with a return of spontaneous circulation (ROSC), and was admitted to the intensive care unit (ICU). He required multiple vasopressors and bicarbonate infusion in addition to several bicarbonate pushes. He was started on continuous renal replacement therapy (CRRT) with a high flux membrane. High dose of pre- and post- filter fluids was used to improve conductive clearance. CRRT was chosen over hemodialysis due to hemodynamic instability. His pH corrected to normal levels with CRRT in less than 24 hours (Table 3). He was transitioned to hemodialysis the following day. His lactic acid and bicarbonate levels improved to normal levels in 48 hours. 

**Table 2:** The patient's CBC on presentation CBC: complete blood count; WBC: white blood cell; RBC: red blood cell; MCV: mean corpuscular volume; RDW: the red cell distribution width

Parameters	Values
WBC (K/uL)	9.5
RBC (M/uL)	3.91
Hemoglobin (g/dL)	11.6
Hematocrit (%)	39
MCV (FL)	99.8
Platelet Count (K/uL)	318
RDW (%)	16.3
Neutrophils (%)	49.5
Band (%)	5.7
Lymphocytes (%)	33.3
Monocytes (%)	1
Metamyelocytes %)	8.6
Myelocytes (%)	1.9
Anisocytosis	1+

**Table 3:** The patient's initial ABG and the much-improved ABG on CRRT in less than 24 hours ABG: arterial blood gas

Parameters	ABG on presentation (intubated)	ABG after 24 hours on CRRT (intubated)
pH Art	<6.69	7.42
pCO_2_	40.4	34.7
pO_2_	138	94
HCO_3_	could not calculate	22.9
Base Excess	could not calculate	1

The patient's urine and blood cultures did not grow any microorganism. Chest x-ray (CXR) did not show any acute cardiopulmonary process. His head computed tomography (CT) did not show any acute abnormality. CT chest abdomen pelvis did not reveal any acute pathology that can explain the patient's severe lactic acidosis. The patient was on metformin 1 gram twice daily and sitagliptin 100 mg daily at home prior to this hospitalization. A blood metformin level was sent on admission and came back as 29 mcg/ml (therapeutic range 1-2 mcg/ml). Methanol, ethanol, ethylene glycol, propylene glycol, and isopropanol levels came back negative (Table 4).

**Table 4:** Toxicology panel showing significantly elevated blood levels of metformin

Toxicology panel	Values	Reference range
Metformin	29	1-2 mcg/mL
Salicylate	<2.5	<2.5
Acetaminophen	<10	<10
Desipramine	<5	Therapeutic range changes
Imipramine	<5	Therapeutic range changes
Amitriptyline	18	Therapeutic range changes
Doxepin	<5	Therapeutic range changes
Doxepin Total	<5	Therapeutic range changes
Nordoxepine	<5	Therapeutic range changes
Total Amitriptyline and Nortriptyline	31	100 - 250 mcg/L
Desmethylclomipramine	<5	150 - 350 mcg/L
Total Clomipramine	None detected	Unknown
Methanol	<0.01	<0.01
Ethylene glycol	20	
Isopropanol	<0.01	<0.01
Acetone	140	<100 = negative, >150 = acetone metabolic imbalance

A review of the patient's electronic medical records revealed that he was started on lisinopril 5 mg daily and amitriptyline 25 mg daily four weeks prior to this admission and had normal kidney function (BUN, creatinine, GFR) at that time. His renal function normalized within four days, and he did not need hemodialysis after that. He was ultimately discharged to an acute rehabilitation facility on day seven of hospitalization.

## Discussion

Metformin is a widely used biguanide anti-diabetic medication with multiple mechanisms of action, which decreases insulin resistance and hepatic glucose output; it enhances peripheral glucose uptake with net effects of decreasing fasting and post-prandial blood glucose [2]. These overall benefits make it the first-line drug as it does not cause hypoglycemia as often as the most other classes of oral anti-diabetic medications. Although generally considered as safe, metformin's most serious but rare side effect is MALA. The mechanism of MALA is complex. Metformin promotes the conversion of glucose to lactate in the splanchnic bed of the small intestine and also inhibits mitochondrial respiratory chain complex 1, leading to decreased hepatic gluconeogenesis from lactate, pyruvate, and alanine resulting in additional lactate and substrate for lactate production. In the absence of acute overdose, metformin-associated lactic acidosis rarely develops in patients without comorbidities such as renal or hepatic insufficiency or acute infection. However, in the rare circumstance when metformin-associated lactic acidosis develops, mortality is high [1,3]. Higher mortality was associated with increased age, lower arterial pH, elevated prothrombin time, and need for mechanical ventilation and vasoactive medications [4].

MALA can occur any time a patient on metformin suffers disruption in renal function resulting in the accumulation of metformin. MALA generally presents with nausea, vomiting, and fatigue, mimicking sepsis [5]. Reportedly, the patient was complaining of nausea, vomiting, and abdominal pain for a few days prior to presentation. Interestingly, he was also complaining of "tunnel vision" to his family. In this case, the patient was found to have a severe anion gap metabolic acidosis with a massively elevated lactic acid despite an acceptable blood pressure, benign abdominal examination, and lack of fever. It is not necessarily due to impaired tissue perfusion, but rather to interference with ATP production. In our patient, the elevation in lactic acid was due, in part, to the accumulation from hepatic gluconeogenesis inhibition and, more importantly, accumulation of NADH (from oxidative phosphorylation inhibition) which results in shunting of pyruvate to lactate (in lieu of acetyl-CoA). It is possible that our patient progressively developed alcoholic ketoacidosis and acute renal failure from dehydration and excessive drinking in the setting of a newly started angiotensin-converting-enzyme (ACE) inhibitor. This could have been the initial insult, but since ethylene glycol toxicity could not be ruled out without levels, he was also treated with fomepizole. If presentation delayed enough, ethylene glycol level might actually be normal given that it is not ethylene glycol itself, which causes toxicity but rather its metabolites glycolic acid and oxalic acid. However, given the history, AKI, and renal failure from dehydration may be more likely. The report of vision changes brought to mind methanol toxicity. However, renal failure is not common. As suspected, blood metformin levels sent during the initial presentation came back significantly elevated at 29 mcg/ml (therapeutic range: 1-2 mcg/ml).

Recommendations for the optimal treatment of MALA mostly depend on expert opinion and case reports. Initial treatment is restricted to supportive measures, although hemodialysis may offer a protective effect [1,5,6]. Metformin is exclusively renally cleared. While metformin is not typically considered a "dialyzable" drug due to its high volume of distribution, there is no other alternative [6]. In our patient, we chose to proceed with CRRT due to significant hemodynamic instability requiring multiple vasopressors at the maximum doses. However, as soon as his hemodynamics improved, he was switched to hemodialysis. Ultimately, in less than 24 hours, his pH improved to normal physiologic limits. Our case demonstrates that even in extreme cases of MALA, prompt and adequate supportive measures can produce a favorable outcome.

## Conclusions

Although rare, MALA can be encountered in the ICU and continues to be a life-threatening condition. In view of current literature, recommendations for the optimal treatment of MALA depend on expert opinion and case reports. Treatment is restricted mostly to supportive measures, although hemodialysis may possess a protective effect. Our case demonstrates that even in extreme cases of MALA, with the prompt and adequate supportive measures, a favorable outcome is possible. More studies with larger patient populations are needed to reach definitive and data-driven recommendations on the treatment of MALA.
